# Molecular alterations in MAPK/ERK, β-Catenin/Wnt, and PI3K/mTOR pathways in adenomatoid odontogenic tumor

**DOI:** 10.1590/1678-7765-2026-0073

**Published:** 2026-06-19

**Authors:** Jintana PANKAM, Puangwan LAPTHANASUPKUL, Nakarin KITKUMTHORN, Siribang-on Piboonniyom KHOVIDHUNKIT

**Affiliations:** 1 Mahidol University Faculty of Dentistry Bangkok Thailand Mahidol University, Faculty of Dentistry, Bangkok, Thailand.; 2 Mahidol University Faculty of Dentistry Department of Oral and Maxillofacial Pathology Bangkok Thailand Mahidol University, Faculty of Dentistry, Department of Oral and Maxillofacial Pathology, Bangkok, Thailand.; 3 Mahidol University Faculty of Dentistry Department of Oral Biology Bangkok Thailand Mahidol University, Faculty of Dentistry, Department of Oral Biology, Bangkok, Thailand.; 4 Burapha University Faculty of Dentistry Chon Buri Thailand Burapha University, Faculty of Dentistry, Chon Buri, Thailand.; 5 Mahidol University Faculty of Dentistry Department of Advanced General Dentistry Bangkok Thailand Mahidol University, Faculty of Dentistry, Department of Advanced General Dentistry, Bangkok, Thailand.

**Keywords:** Adenomatoid odontogenic tumor, KRAS, CTNNB1, PIK3CA, β-catenin, p-mTOR, p-ERK1/2

## Abstract

**Background:**

Previous studies have suggested that tumorigenesis of adenomatoid odontogenic tumor (AOT) may involve the activation of the mitogen-activated protein kinase/extracellular signal-regulated kinase (MAPK/ERK) pathway. However, research on other oncogenic signaling pathways in AOT remains limited.

**Objective:**

This study aimed to investigate gene mutations (Kirsten rat sarcoma viral oncogene homolog (*KRAS*)*,* catenin beta 1 (*CTNNB1*)*,* and phosphatidylinositol-4,5-bisphosphate 3-kinase catalytic subunit alpha (*PIK3CA*)) and their associated protein expressions (phosphorylated extracellular signal-regulated kinase 1 and 2 (p-ERK1/2), β-catenin, and phosphorylated mechanistic target of rapamycin (p-mTOR)) in AOT cases. The association between gene mutations and protein expression was also examined.

**Methodology:**

In total, eight formalin-fixed, paraffin-embedded AOT tissue samples were manually micro-dissected for DNA extraction. Polymerase chain reaction was performed. Positive samples underwent DNA sequencing. Mutations were analyzed in *KRAS* exon 2 (codons 12 and 13), *CTNNB1* exon 3 (codons 32-45), and *PIK3CA* exon 9 (codons 542-549). Protein expression was assessed using immunohistochemistry.

**Results:**

*KRAS* mutations (specifically G12R and G12V) were detected in three of the eight cases (37.5%). *CTNNB1* mutation (T42I) was identified in one of six cases (16.7%), whereas no *PIK3CA* mutation was observed. Moreover, moderate expression of p-ERK1/2 and p-mTOR were found in tumor cells of AOT. β-catenin accumulation was detected in seven cases (87.5%), predominantly showing membranous and cytoplasmic localization with no nuclear β-catenin expression. Statistical analysis indicated no association between gene mutations and protein expression.

**Conclusions:**

*KRAS* mutations and p-ERK1/2 expression support a potential role of MAPK/ERK signaling in AOT pathogenesis. The absence of *PIK3CA* mutations despite p-mTOR expression may in part suggest mutation-independent activation of the PI3K/mTOR pathway. The lack of nuclear β-catenin accumulation may suggest that canonical Wnt signaling is less likely to significantly contribute to AOT tumorigenesis. Further studies with larger cohorts and investigations of additional molecules related to these pathways are warranted.

## Graphical abstract

**Figure:**
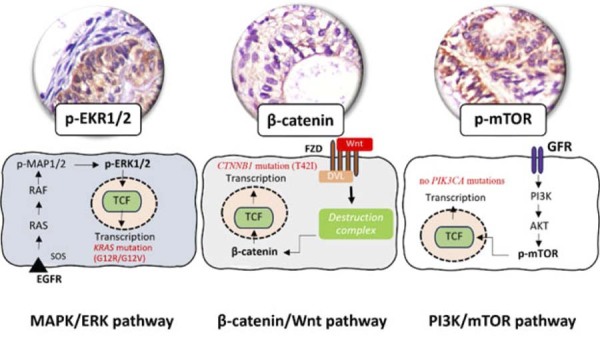

## Introduction

Adenomatoid odontogenic tumor (AOT) is categorized as epithelial odontogenic tumors due to its histomorphological resemblance to components of the dental organ. AOT is a benign odontogenic tumor that is typically considered non-invasive and non-aggressive, displaying slow but progressive growth.^1,2^ Despite being generally regarded as an uncommon tumor, AOTs frequently occur during the second decade of life, with a greater prevalence in young women. This tumor commonly manifests in the maxilla, particularly affecting the anterior jaw bone.^3,4^

Previous studies have suggested that the activation of the mitogen-activated protein kinase/extracellular signal-regulated kinase (MAPK/ERK) signaling pathway is associated with AOT pathogenesis via Kirsten rat sarcoma viral oncogene homolog (*KRAS*) mutations (G12V and G12R).^5,6^ Extracellular signal-regulated kinase 1/2 (ERK1/2) is a member of the mitogen-activated protein kinase (MAPK) family, which mediates signaling cascades that transmit extracellular signals to intracellular targets.^7^ The ERK cascade involves Ras/Raf proteins, MEK1/2, ERK1/2, and downstream mitogen-activated protein kinase-activated protein kinases.^7,8^ Dysregulation of ERK signaling, particularly excessive activation of upstream proteins and kinases, has been implicated in cancer, inflammation, and developmental abnormalities.^7,9^ Importantly, driver mutations in *Ras* (mainly *KRAS*) are the most common mutations in cancer, appearing in approximately 30% of all cancer types.^10^ However, the association between *KRAS* mutations and associated protein expression has not been found in AOT.^11^

Additionally, strong cytoplasmic accumulation of β-catenin protein has been reported in AOT.^12,13^ This protein, encoded by the catenin beta1 *(CTNNB1)* gene, is a central component of the β-catenin/Wnt signaling pathway, which involves the nuclear translocation of β-catenin and the activation of target genes via T-cell factor/lymphoid enhancer-binding factor (TCF/LEF) transcription factors. The activation of this pathway is characterized by the cytoplasmic–nuclear shuttling of β-catenin.^14^ Previous studies have reported nuclear accumulation of β-catenin in many cancers, including malignant odontogenic tumors.^13,15,16^ Alterations of the *CTNNB1* gene and other genes associated with the Wnt signaling pathway have also been investigated in ameloblastoma, calcifying odontogenic cyst, various malignant odontogenic tumors, and other cancers.^13,17,18^ These studies suggest that β-catenin may serve as a useful diagnostic factor in tumorigenesis.^13^ However, investigations of β-catenin expression and *CTNNB1* mutations in AOT remain inconclusive due to limited publications and the absence of detectable *CTNNB1* mutations.^12,13^

Interestingly, the activation of the PI3K/mTOR signaling pathway is yet to be investigated in AOT. The phosphatidylinositol-4,5-bisphosphate 3-kinase catalytic subunit alpha (*PIK3CA*) mutation constitutes one of the most commonly mutated oncogenes across tumor lineages.^19^ *PIK3CA* mutations have been found in head and neck squamous cell carcinoma, gastric cancer, and gallbladder cancer.^19-21^ Moreover, next-generation sequencing has detected *PIK3CA* mutations in odontogenic myxoma.^22^ In normal condition, mechanistic target of rapamycin (mTOR) regulates protein biogenesis by stimulating p70 ribosomal S6 kinase (an enhancer of mRNA translation) and by inhibiting eukaryotic translation initiation factor 4E-binding protein 1 (a translational repressor).^23^ Previous immunohistochemical analyses have shown that about 87% of ameloblastoma cases expressed mTOR.^24^ It has been suggested that investigating the PI3K/mTOR signaling pathway may provide a valuable approach for elucidating pathogenesis, determining aggressiveness, and guiding the selection of optimal therapeutics.^24^

The number of studies assessing the genetic alterations in AOT remains limited, providing insufficient evidence to draw conclusions regarding the relationship between gene and protein alterations and its clinical behavior.^6,11^ Therefore, this study aimed to investigate oncogene mutations including *KRAS*, *CTNNB1*, and *PIK3CA* and the expression of p-ERK1/2, β-catenin, and p-mTOR proteins associated with the MAPK/ERK, β-catenin/Wnt, and PI3K/mTOR pathways, respectively. The association between gene mutations and their corresponding protein expressions was also evaluated. These results may enhance our understanding of the genetic background of this odontogenic tumor.

## Methodology

### Human ethic approval and sample selection

This study received ethical approval from the Institutional Review Board of the Faculty of Dentistry and the Faculty of Pharmacy at Mahidol University (COA No. MU-DT/PY-IRB 2023/071.1411). Informed consent was waived because archived specimens were used and no identifiable patient information was accessed. A total of eight formalin-fixed paraffin-embedded AOT tissues and their demographic data were collected from the Department of Oral and Maxillofacial Pathology at the Faculty of Dentistry, Mahidol University, Thailand, covering cases from 2006 to 2022. Eligible samples meeting specific criteria included reconfirmation of the pathological diagnosis by a board-certified oral pathologist (PL), collection by incisional or excisional biopsies, and a tumor cell composition of at least 80%. Samples lacking sufficient tumor area or optimal DNA quality were excluded.

### Tumor tissue collection for DNA isolation using a manual technique

AOT tissue sections of approximately five microns in thickness were prepared on slides, with at least five sections per sample. Hematoxylin and eosin-stained slides (H&E slides) of all samples were observed under microscope for labeling tumor cells. Only tumor cells were collected after mapping the tumor tissue of H&E with prepared sample slides. Approximately 90% of tumor tissue was scraped by a 0.5 cm stainless spatula and transferred to a 1.5-ml tube for DNA extraction. The DNA was isolated using phenol-chloroform extraction. The resulting DNA pellets were resuspended in 30-50 μl of distilled water. DNA quantity was then assessed using a NanoDrop spectrophotometer (Thermo Scientific™, ND2000USCAN, USA). A concentration of approximately 100 ng/μl with an optimal purity ratio of 1.8 at OD 260/280 nm was used for gene amplification.

### Genes amplification by polymerase chain reaction (PCR)

Glyceraldehyde-3-phosphate dehydrogenase *(GAPDH)* gene amplification was conducted as a quality control measurement for the DNA samples. The PCR reaction mixture, prepared with various primers, consisted of the following components: 0.25 µM primer, 1× buffer, 2 mM MgCl₂, 10 ng/µl DNA sample, 0.2 mM deoxyribonucleotide triphosphates (dNTPs), and 0.5 units of Taq DNA polymerase (Thermo Scientific™, Vilnius, Lithuania). Primer sequences and PCR conditions are listed in Table 1, with 40 PCR cycles performed. Fibrous tissue DNA and sterile distilled water served as positive and negative controls, respectively. PCR products were analyzed by electrophoresis on 1% agarose gel stained with Intron RedSafe™ (iNtRON, South Korea) and visualized under UV transillumination using a GelDoc system (Bio-Rad^®^, CA, USA).

**Table 1:** The primer sequences and conditions for amplifying target DNA

Primers	Sequences	Annealing temperature	DNA size
KRAS	Exon 2	58 ^o^C	157 bp
	F- 5’- AAAGGTACTGGTGGAGTATTTGA-3’		
	R- 5’ -TCAAGGCACTCTTGCCTACG-3’		
CTNNB1	Exon 3	60 ^o^C	216 bp
	F-5’-GATTTGATGGAGTTGGACATGG-3’		
	R-5’-TGTTCTTGAGTGAAGGACTGAG-3’		
PIK3CA	Exon 9	58 ^o^C	196 bp
	F-5’-CCAGAGGGGAAAAATATGACA-3’		
	R-5’-CATTTTAGCACTTACCTGTGAC-3’		
GAPDH	F-5’-TGAGGCTCCCACCTTTCTCATC-3’	56 ^o^C	157 bp
	R-5’-TGAGGCCCTGCAGCGTACTC- 3’		

### DNA sequencing and gene mutation analysis

Positive PCR products were sent to Macrogen Company in South Korea for DNA sequencing. DNA sequencing results were analyzed on BioEdit for comparisons with reference gene sequences in the National Center for Biotechnology Information, specifically sequences IDs NG_007524.2, AY081165.1, and NG_012113.2 for *KRAS*, *CTNNB1,* and *PIK3CA*, respectively. Electropherograms were manually examined to find mutations in *KRAS* exon 2 codons 12 and 13,^25^ *CTNNB1* exon 3 codons 32 to 45,^26^ and *PIK3CA* exon 9 codons 542 to 549.^20^

### Protein expression by immunohistochemistry

All sample slides were prepared with three-micron thickness for investigation. Briefly, sample slides were deparaffinized and rehydrated using xylene and a series of graded ethanol concentrations (100, 95, 80, and 70%). To induce antigen epitope retrieval, the slides were heated with 1× Target Retrieval Solution (pH 6.8; S1699, Dako, Santa Clara, USA) for 12 minutes, followed by blocking of endogenous peroxidase activity with 3% H₂O₂ for 10 minutes. The section slides were then incubated with the primary antibody in a humidified chamber at 4°C overnight. Primary antibodies were diluted in Diluent Solution (S0809, Dako, Santa Clara, USA) at the following concentrations: 1:100 for p-ERK1/2 (#4376, Cell Signaling Technology, Inc.), 1:50 for β-catenin (IR702, Dako^®^, Glostrup, Denmark), and 1:100 for p-mTOR (#2976, Cell Signaling Technology, Inc.). Next, the section slides were incubated with Envision (K5007, Dako, Glostrup, Denmark) for 30 minutes at room temperature in a humidified chamber. The color reaction was developed using the Liquid DAB+ substrate chromogen system (K5007, Dako, Glostrup, Denmark), and the section slides were counterstained with Mayer’s hematoxylin (05-06002/L; Bio-Optica Milano Spa, Milano, Italy). Finally, the slides were dehydrated using a series of graded ethanol concentrations (70, 95, and 100%) and mounted with a mounting medium (05-BMHM508, Bio-Optica Milano Spa., Milan, Italy). For the negative control, the primary antibody was omitted and replaced with antibody diluent solution to confirm the absence of nonspecific staining. Positive controls for p-ERK1/2, β-catenin, and p-mTOR were oral squamous cell carcinoma, fibroma with normal covering mucosa, and invasive ductal carcinoma (a form of breast cancer), respectively. The control slides were included in each run to validate the antibodies.

### The interpretation method for immunohistochemistry

All immunohistochemically stained slides were captured at 400× magnification, with three to five randomly selected tissue areas photographed using a digital camera microscope. The immunohistochemical staining images were independently evaluated by two investigators (JP and PL). The expression of p-ERK1/2 and p-mTOR in the nucleus and/or cytoplasm of tumor cells was evaluated semi-quantitatively. The immunoreactive score (IRS) was calculated by multiplying the percentage positivity score (0 = 0%, 1 = 1-9%, 2 = 10-50%, 3 = 51-80%, 4 = 81-100%) by the intensity score (0 = negative, 1 = mild, 2 = moderate, 3 = strong). The final IRS was interpreted as follows: 0-1 (negative), 2-3 (mild), 4-8 (moderate), and 9-12 (strong).^27^ Interobserver agreement was assessed using the Kappa test, indicating strong agreement at 0.825. β-catenin localization was assessed based on the presence or absence of immunostaining in the nucleus, cytoplasm, or membrane.^28^

### Statistical analysis

This study employed PASW Statistics, version 18.0.0 (formerly SPSS Statistics), and WinWrap^®^ Basic for its statistical analyses. Descriptive statistics were used to examine demographic data, gene mutation and the associated protein expression frequencies. The association between gene mutation status (wild-type versus mutated gene) and associated protein expressions was analyzed using Fisher’s exact test.

## Results

### Demographic data and genes amplification by PCR

According to Table 2, AOT was most observed in women (5/8; 62.50%) aged from seven to 38 years, predominantly located in the mandible (5/8; 62.50%). Tumor size ranged from 1.5 to 5.5 cm, typically presenting as intraosseous and well-defined with unilocular radiolucency. Histologically, all specimens showed the follicular subtype. PCR amplification was technically successful for *GAPDH, KRAS*, and *PIK3CA* in all tested samples, whereas *CTNNB1* was successfully amplified in only six out of eight cases.

**Table 2:** Demographic data and gene amplification

No.	Sex	Age (year)	Tumor location	Tumor size (highest dimension in cm)	Radiography	Histological subtypes	*KRAS*	*CTNNB1*	*PIK3CA*
1	Male	11	Maxilla	2.5	Well-defined, unilocular	Follicular	+	+	+
					radiolucency				
2	Female	7	Mandible	1.5	Well-defined, unilocular	Follicular	+	-	+
					radiolucency				
3	Female	31	Mandible	2.5	Well-defined, unilocular	Follicular	+	+	+
					radiolucency				
4	Female	38	Maxilla	5.5	Well-defined, unilocular	Follicular	+	+	+
					radiolucency				
5	Female	22	Mandible	4.7	Well-defined, unilocular	Follicular	+	+	+
					radiolucency				
6	Female	11	Mandible	2.9	Well-defined, unilocular	Follicular	+	+	+
					radiolucency				
7	Male	25	Maxilla	4.1	Well-defined, unilocular	Follicular	+	+	+
					radiolucency				
8	Male	15	Mandible	5.5	Well-defined, unilocular	Follicular	+	-	+
					radiolucency				

Note: +; Positive, and -; negative for PCR amplification.

### Evaluation of oncogene mutations in AOT

**Figure 1:**
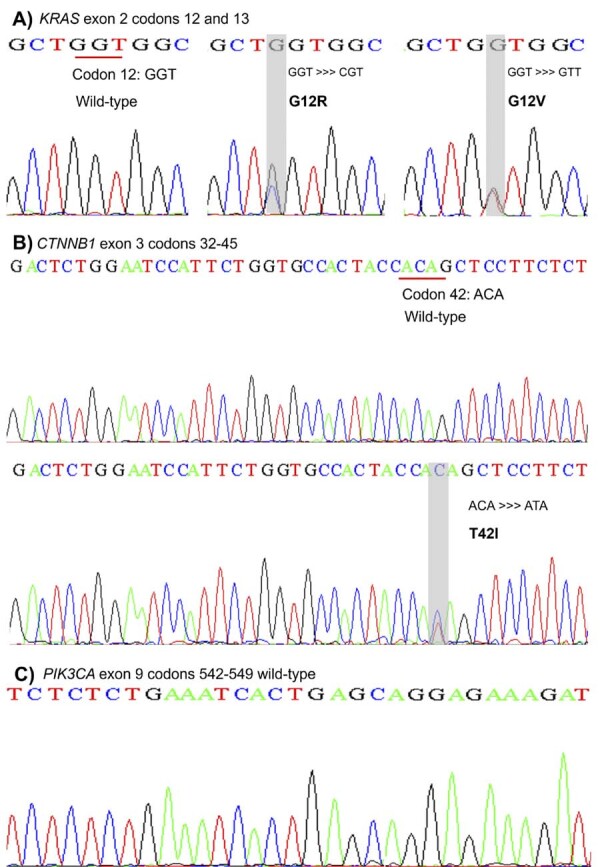
Electropherogram of gene mutation analysis in AOT: A): *KRAS* exon 2 codons 12 and 13 showing wild-type and mutated sequences. B): *CTNNB1* exon 3 codons 32-45 displaying wild-type and mutated sequences. C): *PIK3CA* exon 9 codons 542-549 showing the wild-type sequence. Mutation points are highlighted in gray.

Of the eight AOT cases, *KRAS* wild-type and *KRAS* mutations were observed in 62.50% (5/8) and 37.50% (3/8) of cases, respectively. The identified *KRAS* mutations included G12R in two cases and G12V in one case (Figure 1A). These mutations substitute glycine (G) with arginine (R) and valine (V) at codon 12 of exon 2, respectively. Although most AOT cases (5/6; 83.33%) exhibited wild-type *CTNNB1*, one case showed *CTNNB1* exon 3 codon 42 mutation (T42I), as shown in Figure 1B. This mutation substitutes threonine (T) with isoleucine (I) at codon 42 of exon 3. No mutation was detected in *PIK3CA* exon 9, with all eight cases showing the wild-type sequence (Figure 1C).

### Protein expression in AOT

**Figure 2:**
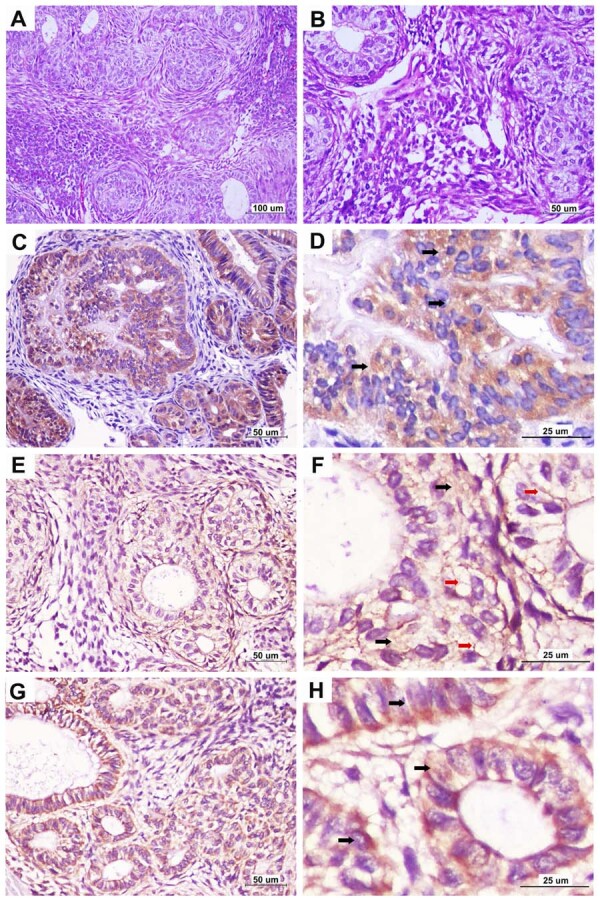
Protein expression in AOT: (A and B) H&E staining showing the histopathological features of AOT at 200× and 400× magnifications, respectively. C (400×) and D (blow-up picture of C): p-ERK1/2 expression in the cytoplasm of tumor cells. E (400×) and F (blow-up picture of E): β-catenin localized in both the cytoplasm and membrane. G (400×) and H (blow-up picture of G): p-mTOR expression in the cytoplasm of tumor cells. Cytoplasmic immunostaining is indicated by black arrows, whereas membranous staining is indicated by red arrows in the higher-magnification images.

The histopathological features of AOT consist of spindle-shaped, cuboidal, or columnar epithelial cells in sheets, nests, duct-like structures and rosette-like formations (Figure 2A and 2B). p-ERK1/2 expression was observed in 87.50% (7/8) of AOT cases. Most cases showed moderate immunostaining (4/7; 57.14%), followed by strong (2/7; 28.57%) and mild (1/7; 14.29%) staining, with a mean IRS ± SD of 6.36 ± 2.86 (Table 3). This protein was mainly observed in the cytoplasm of tumor cells (Figure 2C and 2D) and occasionally in the nucleus of the tumor cells (Figure 2C). The distribution of p-ERK1/2 immunostaining varied, occurring in several structures such as rosettes, ducts, and double-layered spheres. Additionally, β-catenin expression was detected in 87.50% (7/8) of AOT cases, whereas one case lacked this protein (Table 3). β-catenin was predominantly localized in the cytoplasm and membrane of tumor cells (Figure 2E and 2F), although one case only showed cytoplasmic positivity. The expression of β-catenin was mild and scattered within tumor cells. Notably, AOT cases showed no nuclear localization of β-catenin. Furthermore, p-mTOR expression was detected in all AOT cases (100%), with staining primarily in the cytoplasm of tumor cells (Figure 2G and 2H). Regarding p-mTOR expression levels (Table 3), most AOT cases commonly showed moderate expression (6/8; 75.00%), followed by strong expression (2/8; 25.00%), with a mean IRS ± SD of 7.82 ± 1.79. Mild immunostaining for p-mTOR was also present in stromal and endothelial cells.

**Table 3:** Gene mutations and associated protein expression in AOT

No	Gene mutation			Protein expression				
	*KRAS*	*CTNNB1*	*PIK3CA*	p-ERK1/2	β-catenin			p-mTOR
				(IRS)	N	C	M	(IRS)
1	Wt	Wt	Wt	Moderate (8.00)	-	+	-	Moderate (6.10)
2	Wt	-	Wt	Strong (9.18)	-	+	+	Moderate (8.75)
3	**G12R**	Wt	Wt	Mild (3.26)	-	-	-	Moderate (6.00)
4	**G12R**	Wt	Wt	Negative (1.39)	-	+	+	Moderate (6.20)
5	Wt	Wt	Wt	Strong (9.00)	-	+	+	Moderate (7.04)
6	Wt	Wt	Wt	Moderate (6.06)	-	+	+	Strong (9.00)
7	Wt	**T42I**	Wt	Moderate (5.50)	-	+	+	Strong (11.04)
8	**G12V**	-	Wt	Moderate (8.48)	-	+	+	Moderate (8.40)

Note: Wt; wild-type, gene mutation was labeled in bold, -; Negative for PCR amplification or absence of protein expression, +; Positive for PCR amplification or presence of protein expression. p-ERK1/2 and p-mTOR expression levels are presented as immunoreactive scores (IRS): 0–1 (negative), 2–3 (mild), 4–8 (moderate), and 9–12 (strong). β-catenin localization was assessed based on the presence (+) or absence (-) of immunostaining in the nucleus (N), cytoplasm (C), or membrane (M).

### The association of gene mutation and protein expression

Table 4 indicates that two of the three *KRAS*-mutant cases (66.67%) and a *CTNNB1* mutation case (100%) expressed p-ERK1/2, β-catenin, and p-mTOR. However, most wild-type cases for all three genes also showed protein expression. Statistical analysis evinced no significant association between gene mutations and protein expression (*p*>0.05).

**Table 4:** The association of gene mutation and protein expression

Expression	*KRAS* (n=8)		*CTNNB1* (n=6)		*PIK3CA* (n=8)	
	Mt	Wt	Mt	Wt	Mt	Wt
	n (%)	n (%)	n (%)	n (%)	n (%)	n (%)
**p-ERK1/2**						
Negative	1 (12.50)	0	0	1 (16.67)	0	1 (12.50)
Positive	2 (25.00)	5 (62.50)	1 (16.67)	4 (66.67)	0	7 (87.50)
**β-catenin**						
Negative	1 (12.50)	0	0	1 (16.67)	0	1 (12.50)
Positive	2 (25.00)	5 (62.50)	1 (16.67)	4 (66.67)	0	7 (87.50)
**p-mTOR**						
Negative	0	0	0	0	0	0
Positive	3 (37.50)	5 (62.50)	1 (16.67)	5 (83.33)	0	8 (100)

Note: Mt; mutation, Wt; wild-type

## Discussion

The mutations of *KRAS* at codons 12, 13, or 61 impaired intrinsic hydrolysis rates or affected binding to GTPase-activating proteins.^29^ Moreover, these mutations can differentially affect the distribution of GTP-bound Ras among conformational states that have varying affinities for effector proteins, altering effector recognition and the downstream signaling pathways of the MAPK/ERK.^30^ In this study, 37.50% of AOT cases showed *KRAS* exon 2 codon 12 mutation (G12R/V), with almost all (2/3) of these tumors being positive for p-ERK1/2. Our findings showed a lower frequency of *KRAS* mutation than previous studies,^6,11^ which reported that more than 70.00% of AOT cases showed *KRAS* mutations. The discrepancy between our findings and those of other studies may be attributed to differences in the techniques used to detect gene mutations. Previous studies performed next-generation sequencing or oncogenic panel assays.^6,11^ Additionally, population differences and the genetic heterogeneity of AOT may also contribute to mutation frequency variability as most previous molecular studies have been conducted in South American cohorts. Evidence from Asian populations remain limited. Only a Chinese cohort reported *KRAS* mutations in approximately 64% of AOT cases,^31^ which is also lower than the frequency in Brazilian cohorts (71%).^6^ Regarding immunohistochemistry, the expression of ERK1/2 and other proteins related to MAPK/ERK signaling pathway in AOT was also observed in almost all of AOT cases.^11^ Taken together, these findings suggest that *KRAS* exon 2 codon 12 mutations may activate the MAPK/ERK signaling pathway in AOT. Additionally, a previous study concluded that a high frequency of *KRAS* exon 2 codon 12 mutation in AOT also occurred independently of clinicopathological characteristics.^6^ Our study found no significant differences in associated protein expression between *KRAS*-mutated and wild-type cases. Its small sample size may have limited its ability to assess the association between *KRAS* mutation status and demographic data. Further studies with a larger sample size should be considered.

We found that most AOT cases showed wild-type *CTNNB1* exon 3, whereas one AOT case had a *CTNNB1* mutation (T42I). This mutation has not been previously documented in published molecular studies of AOT. Missense mutations in *CTNNB1* exon 3 affect regulatory amino acids in the N-terminal region of β-catenin.^18^ An *in silico* analysis using MutationTaster suggested that the p.T42I variant results in an amino acid substitution, possibly affecting protein function and altering splice site regulation. Nevertheless, the biological significance of this mutation remains unclear. Therefore, further investigations, including additional *in silico* pathogenicity prediction analyses and functional studies, are warranted to explain the potential role of *CTNNB1* exon 3 mutation in AOT tumorigenesis.

A previous study has suggested that *CTNNB1* mutations may contribute to nuclear β-catenin accumulation in tumor cells of endometrial cancer, supporting the oncogenic role of aberrantly activated β-catenin in epithelial cells.^16^ However, in our study, β-catenin accumulation was observed in the cytoplasm and membrane of tumor cells in most AOT cases (87.50%), whereas no nuclear accumulation of β-catenin was detected. This result is in line with a previous case report which documented strong cytoplasmic expression of β-catenin in AOT but found no *CTNNB1* exon 3 mutation.^12^ Based on our study, this expression pattern suggests that, although β-catenin is abundantly present, canonical Wnt/β-catenin signaling is unlikely to be fully activated in AOT, as the nuclear translocation of β-catenin configures a critical step for transcriptional activation of Wnt target genes. Membranous β-catenin localization is consistent with its role in cell-cell adhesion via the cadherin-catenin complex, which may reflect the well-differentiated and generally indolent biological behavior of AOT.

Regarding case number seven, *CTNNB1* mutation was found in the absence of nuclear β-catenin immunostaining. Although *CTNNB1* mutations are commonly associated with nuclear accumulation of β-catenin, this correlation is far from absolute. A previous study in endometrial carcinoma has shown that nearly half of cases harboring *CTNNB1* mutations had β-catenin nuclear localization in only 5-10% of tumor cells.^16^ Moreover, the extent of nuclear β-catenin expression showed no correlation with specific *CTNNB1* mutations. Kim and colleagues suggested that the subcellular localization of β-catenin is context-dependent and may vary according to tumor type, degree of differentiation, and microenvironmental factors.^16^ These findings indicate that the absence of nuclear β-catenin staining fails to exclude the presence of *CTNNB1* mutation, and molecular testing remains the gold standard for definitive assessment.

The absence of nuclear β-catenin accumulation in all cases in our study supports the notion that dysregulated Wnt signaling may not play a dominant oncogenic role in AOT pathogenesis. These findings agree with a previous study reporting limited or absent nuclear β-catenin expression in odontogenic tumors with benign clinical behavior.^12^ However, the small sample size of our study prohibited the complete exclusion of the involvement of the Wnt signaling pathway in AOT tumorigenesis. Other regulatory mechanisms of the non-canonical Wnt pathway may also be involved. Further studies are required to investigate other molecular alterations in this pathway that may contribute to the development of AOT.

A previous study has reported that *PIK3CA* mutations, particularly in exons 9 (helical domain) and 20 (kinase domain), occur in approximately 80% of solid tumors and that they can impact protein structure. These mutations have been associated with shortened disease-specific survival, increased cell invasion, and metastasis.^21^ The activation of the PI3K/mTOR signaling pathway was studied in dentigerous cysts, odontogenic keratocysts, and ameloblastoma.^32^ More than 70.00% of these lesions expressed p-AKT protein, suggesting that the PI3K/mTOR pathway may play a role in the pathogenesis of these odontogenic lesions.^32^ To our knowledge, this study is the first to evaluate the involvement of the PI3K/mTOR signaling pathway in AOT. All examined AOT cases showed wild-type *PIK3CA* exon 9, accompanied by moderate p-mTOR expression. This finding suggests that mTOR activation in AOT may occur independently of *PIK3CA* exon 9 mutations. Similar observations have been reported in esophageal carcinoma, in which p-mTOR expression was detected in the absence of *PIK3CA* mutations,^33^ as well as in breast cancer, in which mTOR activation was insignificantly associated with *PIK3CA* mutation status.^34^ These findings imply that mTOR activation can occur by alternative upstream mechanisms rather than by only depending on *PIK3CA* mutations. However, it should be noted that mutation analysis in this study was limited to *PIK3CA* exon 9 as we were unable to successfully amplify *PIK3CA* exon 20 from the formalin-fixed paraffin-embedded samples via PCR. Therefore, the possibility of additional mutations in other regions of *PIK3CA* must be considered. The high level of p-mTOR expression should be interpreted with caution because mTOR phosphorylation may be influenced by other signaling pathways or microenvironmental factors. Future studies involving larger sample sizes, analyses of additional *PIK3CA* hotspot regions, and broader genomic approaches including next-generation sequencing would be valuable to further explain the mechanisms underlying PI3K/mTOR pathway activation in AOT and its potential role in tumorigenesis.

## Conclusion

Although the small sample size is a limitation of this study, our findings highlight the potential role of the MAPK/ERK signaling pathway in AOT pathogenesis as evinced by *KRAS* mutations and p-ERK1/2 expression. Furthermore, the absence of nuclear β-catenin accumulation implies that canonical Wnt signaling may be less likely to play a significant role in tumorigenesis in these tumors. Similarly, the overexpression of p-mTOR in the absence of *PIK3CA* mutations suggests the need for further research to elucidate the mechanisms underlying PI3K/mTOR pathway activation in AOT. Further studies with larger cohorts or investigations involving additional molecules related to these pathways are warranted.
